# Influence of Ambient Relative Humidity on the Shrinkage Strain of Engineered Geopolymer Composites Based on Orthogonal Experimental Design

**DOI:** 10.3390/ma17174321

**Published:** 2024-08-30

**Authors:** Hongyin Wang, Yuelong Zheng, Zhenyun Yu

**Affiliations:** 1 School of Civil Engineering, Zhengzhou University, Zhengzhou 450001, China; wanghongyin@zzmetro.cn; 2 Zhengzhou Metro Group Co., Ltd., Zhengzhou 450001, China

**Keywords:** engineered geopolymer composites, orthogonal experiment, key variables, relative humidity, drying shrinkage strain

## Abstract

With the aim to systematically analyze the ambient relative humidity on the shrinkage strain of Engineered Geopolymer Composites (EGCs), this paper studied four variables (fly ash to ground granulated blast furnace slag mass ratio, alkali content, water–binder ratio, and fiber volume content) though orthogonal experimental design and three different relative humidity values (30%, 60%, and 100% RH). The results indicated that, for EGC specimens under 30% RH and 60% RH, the decrease in slag content and increase in alkali content both resulted in greater drying shrinkage. The addition of fibers effectively reduced the shrinkage strain, while a minor impact on shrinkage was presented by the W/B ratio. The first and second key factors affecting the drying shrinkage strain were the FA/GGBS ratio and the alkali content. The optimal ratio of FA/GGBS, alkali content, and fiber volume fraction were 0/100, 4%, and 1.5%, respectively. Dring shrinkage strain was decreased with the increase in ambient relative humidity. Compared with the shrinkage strain under 30% RH, the reduction in shrinkage strain under 60% RH and 100%RH was up to 46.1% and 107.5%, respectively. At last, a relationship between shrinkage strain and curing age under 30% and 60% RH was established with a fitting degree from 0.9492 to 0.9987, while no clear relationship was presented under 100% RH. The results in this paper provide a practical method for solving the shrinkage problem of EGCs.

## 1. Introduction

As a novel green binder material, geopolymer is an inorganic binder prepared by activating aluminosilicate-rich raw materials, such as blast furnace slag and fly ash, with an alkali activator. Its reaction mechanism involves the cleavage of chemical bonds like -Si-O-Si-, -Si-O-Al-, and -Al-O-Al- within the raw materials in a highly alkaline environment, releasing active Si and Al monomers. These monomers undergo polymerization to form a three-dimensional network structure composed of SiO_4_ and AlO_4_ units linked by shared oxygen atoms.

This unique spatial structure enables geopolymer to exhibit the characteristics of high early/late strength, acid resistance, fire resistance, and so on [1,2]. Moreover, when compared with that of Ordinary Portland Cement (OPC), CO_2_ emissions during production of geopolymer binder materials can be reduced by approximately 50% to 80%, with the advantages of environmental friendliness and the ability to reuse solid waste. Despite its performance being comparable to or even superior to OPC, geopolymer inevitably shows a quasi-brittle characteristic similar to those of OPC. And its shrinkage property is of the greatest concern, reaching 2–3 times that of OPC [3]. 

Fiber composite technology is an effective method for enhancing material performance. By incorporating fibers into geopolymers, the ductility is significantly increased, resulting in a new type of fiber-reinforced composite material known as Engineered Geopolymer Composites (EGC). Up to now, the current research on EGC has mainly focused on mechanical and durability properties, while little research exists on shrinkage performance [4,5]. Evaluating shrinkage performance is crucial for assessing durability, as excessive shrinkage can lead to cracks in structures or components, allowing harmful agents from the environment to penetrate and adversely affect structural durability. 

The factors influencing the shrinkage of geopolymers are numerous, including the type and dosage of mineral admixtures, the dosage of alkali activators, the water–cement ratio, and the curing conditions [6,7,8]. Studies have shown that an increase in slag content in fly ash–slag-based polymer concrete leads to increased autogenous shrinkage [9,10], while increasing fly ash content may result in a decrease in strength, but also a reduction in drying shrinkage [11,12]. However, other studies indicated that increasing slag content can reduce shrinkage strain [13,14]. For fly ash–slag-based geopolymer, a higher water–cement ratio leads to a reduction in drying shrinkage, but it has little effect on the drying shrinkage of slag-based geopolymer [15]. When sodium silicate is used as an alkali activator, reducing the modulus of water glass decreases drying shrinkage [16,17]. An increase in alkali dosage may result in greater shrinkage strain [11,18], although some studies indicated that when the alkali dosage exceeds a critical value, shrinkage decreases instead [19]. In addition, cement or geopolymer concrete curing relative humidity both have an important impact on the shrinkage performance, with higher humidity being very effective in alleviating early shrinkage development [7,20,21]. In the EGC system, the addition of fibers can effectively resist shrinkage stress, reduce stress concentration at crack tips, and restrict the development of shrinkage [22,23,24]. The incorporation of steel fiber can reduce the drying shrinkage of fly ash-based geopolymer by about 24% [22]. At a relative humidity of 100%, the maximum strain can be controlled within 1.5 mm/m by adding 0.2 wt % 3 mm or 6 mm long carbon fiber [23]. The addition of polypropylene fibers with volume fractions of 0.05%, 0.1%, 0.15%, and 0.2% can reduce the dry shrinkage strain of alkali-activated slag mortar by 10.3–26.2% [24]. These studies have effectively revealed and clarified the influence of various factors on the shrinkage properties of geopolymer. And the method was mainly focused on single-factor multi-level design. Given the complexity of the hydration reaction of geopolymer binder, many factors affecting experimental results need to be considered, which requires more effort and time to conduct a comprehensive analysis. The orthogonal experimental design method can effectively solve the above problem and analyze the main factors affecting the test results in multiple factors, which is conducive to optimizing the mix ratio design [25,26].

This article aims to systematically analyze the influence of four variables (fly ash to ground granulated blast furnace slag mass ratio, alkali content, water–binder ratio, and fiber volume content) though orthogonal experimental design and three different relative humidity values (30%, 60%, and 100% RH) on the shrinkage strain of Engineered Geopolymer Composites (EGCs). Additionally, it seeks to identify the key factors affecting shrinkage strain, determine the optimal mix ratio, and establish the relationship between shrinkage strain and curing age. The results in this paper would provide a practical method for solving the shrinkage problem of EGCs.

## 2. Experimental Program

### 2.1. Raw Materials

**Figure 1:**
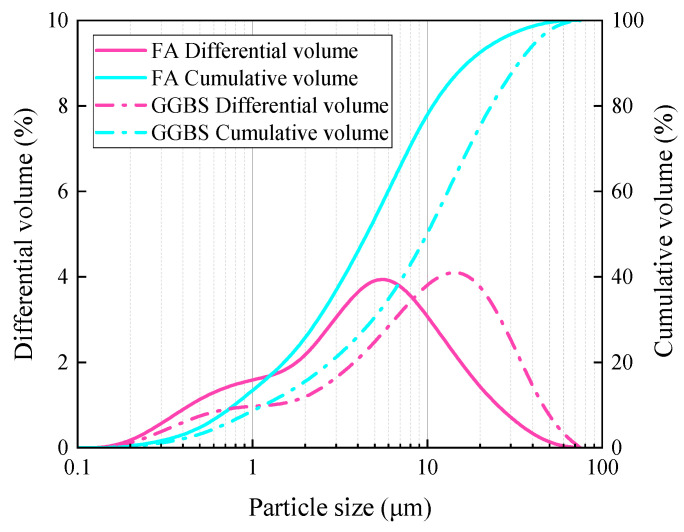
Particle size distribution of FA and GGBS.

Ground granulated blast furnace slag (GGBS) used in this experiment was classified as S95 grade and provided by Changwang Mining Products Co., Ltd. in Shijiazhuang, China. Grade I fly ash (FA) was provided by Yulian Power Plant in Gongyi, China. The chemical composition of FA and GGBS is shown in Table 1, and their particle size distribution is shown in Figure 1.

Alkali activator was prepared by dissolving industrial grade anhydrous sodium metasilicate (Na_2_SiO_3_), a white powder, provided by Henan Huiteng Chemical Technology Co., Ltd. (Luohe, China). Its technical specifications are shown in Table 2.

The PE fibers used in this experiment were produced by Zhejiang Quanmite New Material Technology Co., Ltd. in Jiaxing, China, and their technical specifications are shown in Table 3.

### 2.2. Design of Test Mix Proportion

Orthogonal experiments utilize independent variations and mutually orthogonal experimental matrices, allowing the level of each factor to vary independently. This method can comprehensively analyze the impact of multiple factors on the results in fewer experiments, saving time and resources. Regarding the mass ratio of fly ash to slag, some studies have shown that the incorporation of fly ash will have a favorable impact on inhibiting the shrinkage property of geopolymer cementitious materials [11]. And the higher the slag content, the lower the shrinkage strain [13]. In this experiment, four factors were focused on, namely, the mass ratio of fly ash to slag, the alkali content, the water–binder ratio, and the fiber volume fraction. Each factor was tested at 4 levels, using an $L_{16}(4^{4})$ orthogonal table, as shown in Table 4. The range analysis method was used to determine the influence degree of each factor on the drying shrinkage performance of EGC, and the optimal mix proportion was established.

In addition, the compressive strength shown in Table 4, was tested using cubic specimens with dimensions of 50 mm × 50 mm × 50 mm. Its average value was calculated using three specimens.

### 2.3. Specimen Preparation and Curing

The FA, GGBS, and sodium metasilicate powder were weighed according to the specified proportions and sequentially added to the mixing drum. Initially, the mixture was stirred at low speed for 2 min. Then, the pre-weighed water was added, and the mixture was stirred at high speed for 2 min. Following this, the pre-measured PE fibers were added, and the mixture was stirred for an additional 3 min. The well-mixed mortar was quickly poured into the test mold and placed on a vibrating table for two rounds of vibration. The first round lasted for 45 s, during which the dispersion of fibers was observed. Once evenly vibrated, the surface of the test mold was smoothed with a trowel. After a 10 s interval, the second round of vibration started and lasted for 15 s to ensure uniform compaction of the materials. When casting the drying shrinkage specimen, nail heads must be packed densely on both sides of the test mold. The vibrated sample was wrapped in plastic wrap and then placed in an environment with a temperature of 20 °C ± 5 °C and a relative humidity (RH) of 50% ± 5% RH for 1 day before demolding.

**Figure 2:**
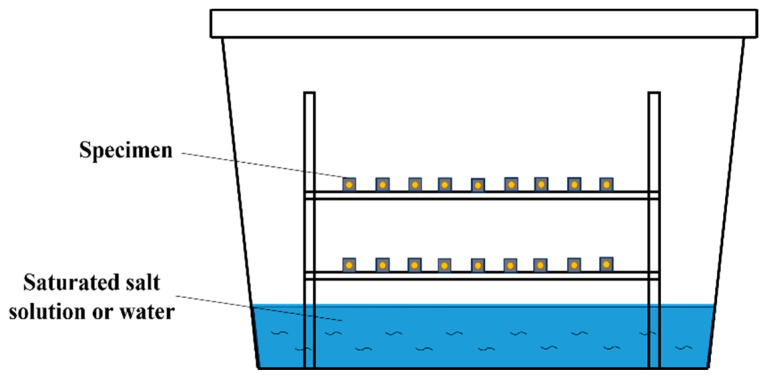
Diagram illustrating the drying shrinkage specimen curing setup.

After demolding, the specimens were placed in a sealed box for curing at a temperature of 20 °C ± 2 °C. Three different relative humidity levels—30%, 60%, and 100% RH—were set to explore their impact on the drying shrinkage of EGCs. The relative humidity was controlled by placing saturated salt solutions at the bottom of the box. Its principle is that at a constant temperature, the increase in salt in the solution will cause a decrease in saturated vapor pressure, resulting in a decrease in relative humidity. Therefore, 30%, 60%, and 100% RH are maintained by the saturated magnesium chloride solution, the saturated sodium bromide solution, and the poured water. The diagram illustrating the drying shrinkage specimen curing setup is shown in Figure 2.

### 2.4. Drying Shrinkage Strain Test

**Figure 3:**
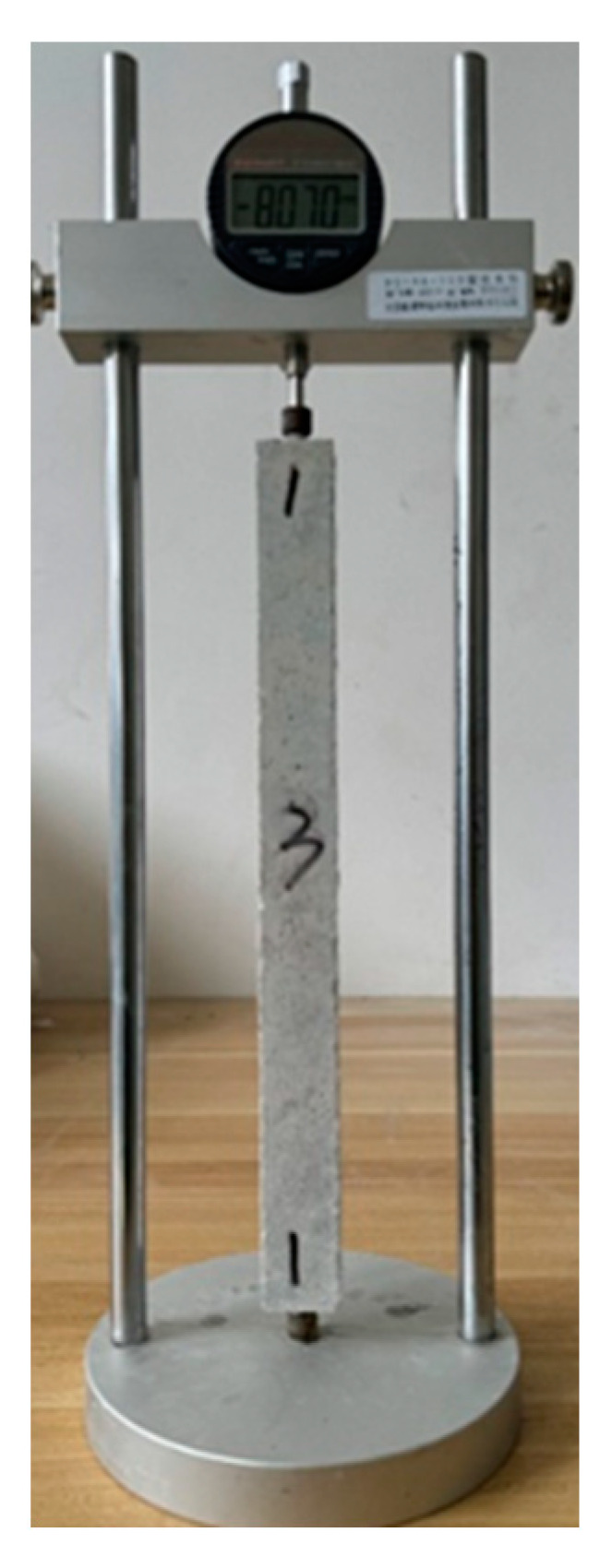
BC156-300 type comparative length gauge.

The drying shrinkage test method followed the Chinese building materials industry standard “Test Method for Drying Shrinkage of Cement Mortar” (JC/T 603-2004) [27]. The length of the specimens was measured using the BC156-300 type comparative length gauge, produced by Beijing Hangjian Huaye Technology Development Co., Ltd. (as shown in Figure 3). This device consists of a digital dial indicator, a bracket, and a calibration rod. The digital dial indicator has an accuracy of 0.001 mm and a range of 12.7 mm.

**Figure 4:**
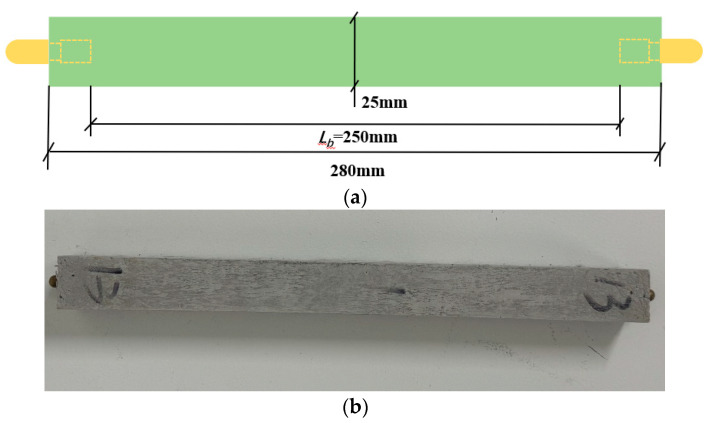
Drying shrinkage specimen: (**a**) schematic diagram, (**b**) test specimen.

The dimensions of the prismatic specimen for shrinkage strain tests were 25 mm × 25 mm × 280 mm, as shown in Figure 4. Each group consisted of three replicated specimens, whose preparation and curing process is presented in Section 2.3. After demolding, the length of the specimen was measured by an elongation comparator and recorded as the initial length, which was then recorded every 24 h.

The shrinkage strain was the average value of three identical specimens, and its calculation formula is shown in Equation (1):

(1) $$\varepsilon_{t}=\frac{L_{t}-L_{0}}{L_{b}}$$

where:

$L_{0}$—The initial length of the specimen, mm;

$L_{t}$—The length of the specimen at time *t*, mm;

$L_{b}$—The effective length of the tested specimen, taken as 250 mm;

$\varepsilon_{t}$—The shrinkage strain value at time *t*, 10^−6^.

## 3. Results and Discussion

### 3.1. Drying Shrinkage Strain

**Figure 5:**
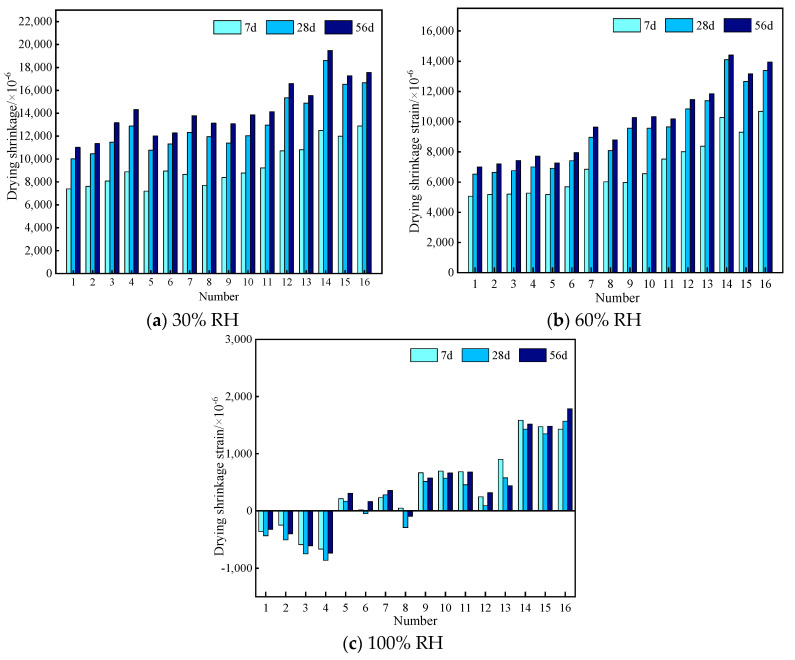
Column chart of the dry shrinkage strain under different relative humidity levels and various ages.

The drying shrinkage strain of EGC specimens at 7 d, 28 d, and 56 d under 30%, 60%, and 100% RH are presented in Table 5, Table 6 and Table 7. Figure 5 shows the column chart of the dry shrinkage strain under different relative humidity levels and various ages. According to these shrinkage strain data, for specimens under 30% and 60% RH, a consistent pattern with increasing age was presented. And their shrinkage strain developed very fast at an early curing age, in which the shrinkage strain of all groups reached more than 55% of the final shrinkage value (the 56 d shrinkage strain was used as the final shrinkage value), and that of some groups reached more than 70%. Subsequently, the shrinkage rate gradually slowed down and stabilized by around 28 d. However, for specimens under 100% RH, the above phenomenon was not observed.

### 3.2. Analysis of Key Factors and Optimal Proportion for Drying Shrinkage

Due to the limitations of visual analysis to account for the impact of various factors, range analysis was performed, which is a statistical method used to evaluate the influence degree of various factors on results. This method quantifies the impact of each factor by comparing the ranges at different factor levels, with the goal of identifying which factors have a significant effect on performance. By calculating the range (denoted as R) for each factor, the relative importance of each factor was determined, in which the larger the R value, the greater the impact of the factor’s level changes on the experimental results. The formula for calculating the R value is shown in Equation (2).

(2) $$R_{j}=max\left(\overset{¯}{K_{j1}},\overset{¯}{K_{j2}},\cdots ,\overset{¯}{K_{jm}}\right)-min\left(\overset{¯}{K_{j1}},\overset{¯}{K_{j2}},\cdots ,\overset{¯}{K_{jm}}\right)$$

where $K_{jm}$ is the sum of the test indexes corresponding to the factor m level in column *j*, and $\overset{¯}{K_{jm}}$ is the average value of $K_{jm}$.

(1) 30% relative humidity

The results of the range analysis on the drying shrinkage strain of EGC specimens under 30% RH are shown in Table 8. Based on the magnitude of R values, it was observed that the R value of the FA/GGBS ratio was significantly larger than the other three factors, indicating its most significant impact on drying shrinkage, while an opposite phenomenon was observed on the R value of the W/B ratio. In a word, the order of factors influencing drying shrinkage at 7 d, 28 d, and 56 d, from strongest to weakest, was as follows: FA/GGBS ratio, alkali content, fiber volume content, and W/B ratio. The key factors were the FA/GGBS ratio and alkali content.

**Figure 6:**
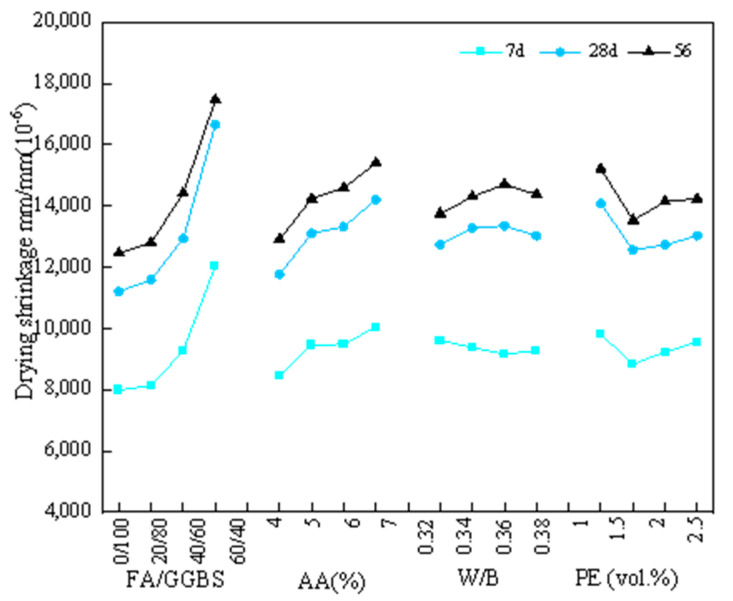
Relationship between the average drying shrinkage strain at different ages and various factors under 30% RH.

Figure 6 presents the relationship between the average drying shrinkage strain at different ages and various factors under 30% RH. Based on the average value K_avg_, it was shown that the drying shrinkage strain at different ages increased with higher FA/GGBS ratio and alkali content. This phenomenon could be attributed to the following reasons: (1) Internal structural compactness and water loss rate, as for an FA/GGBS ratio of 0/100, a dense structure was formed by the higher reactivity and hydration degree of slag, and thus reduced the water loss rate, resulting in the lowest drying shrinkage strain shown in Figure 6. The opposite phenomenon was observed in the specimen with the highest FA content, such that for an FA/GGBS ratio of 40/60, a more loose structure was formed by the lower reactivity and hydration degree of fly ash, and thus increased the water loss rate [28], leading to the highest drying shrinkage strain shown in Figure 6. (2) High alkali content, as for an alkali content of 7%, the increase in alkali content effectively improved the alkalinity of the reaction environment, promoted the dissolution of aluminosilicate in slag and fly ash, and enhanced the dissolution rate of Si and Al [29]. A highly alkaline environment can destroy the aluminosilicate shell formed by the initial hydrolysis of the slag surface, promoting the hydration reaction [30] and improving the compactness of the hydration product structure. This results in an increase in the proportion of pores (2.5 nm–50 nm), leading to higher capillary pressure [31,32]. Furthermore, the high alkali content caused the pore solution to contain a large amount of Na^+^, Ca^2+^, Si^4+^, and Al^3+^ ions, increasing the surface tension of the pore solution [33]. The rapid reduction in these liquid ions will generate large shrinkage stress within the matrix [34,35].

In addition, as for the fiber volume fraction, as shown in Figure 6, the average value K_avg_ at different ages was initially decreased and then increased from 1.0% to 2.5%, presenting an optimal fiber volume content of 1.5%. The reduction in drying shrinkage strain was mainly due to the uniform and disoriented distribution of fibers in the matrix [36]. This not only improved the tensile properties of the matrix itself, but also limited the contraction stress of EGCs due to the interfacial bonding force between the fiber and the matrix [37]. As for the W/B ratio, two trends were present at different ages, and drying shrinkage strain within a narrow range from 0.32 to 0.38 was observed.

Based on the range analysis in Table 8 and Figure 6, the key factors were determined to be the FA/GGBS ratio and alkali content, which was consistent with the existing research [10,11,13,18,32]. The change in these two factors will have a greater impact on drying shrinkage. As for the EGC specimens under 30% RH, according to the minimum shrinkage strain, the optimal FA/GGBS ratio, alkali content, W/B ratio, and fiber volume fraction were determined to be 0/100, 4%, 0.32, and 1.5%, respectively. This corresponded to the A_1_B_1_C_1_D_2_ group.

(2) 60% relative humidity

The results of the range analysis on the drying shrinkage strain of EGC specimens under 60% RH are presented in Table 9. According to the magnitude of R values, a similar trend to that of EGC specimens under 30% RH was present. And the order of factors influencing drying shrinkage at 7 d, 28 d, and 56 d, from strongest to weakest, was as follows: FA/GGBS ratio, alkali content, fiber volume content, and W/B ratio.

**Figure 7:**
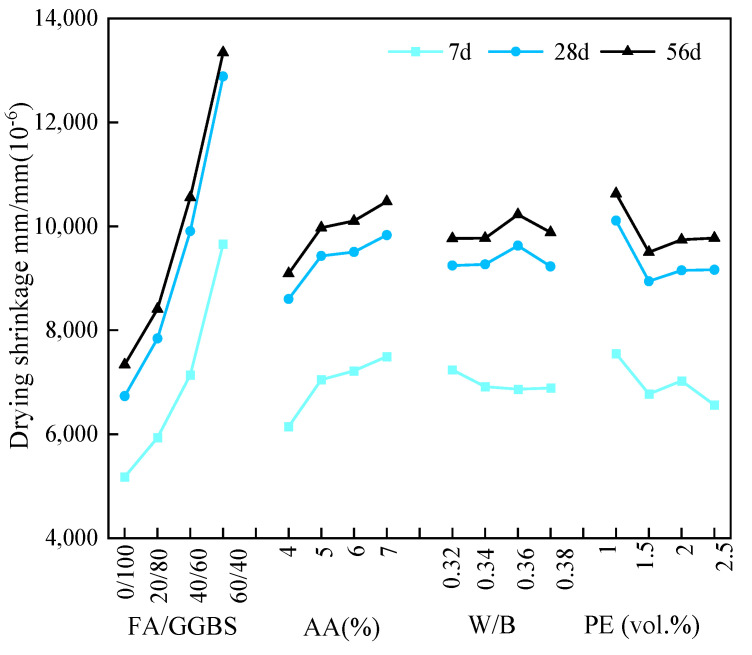
Relationship between the average drying shrinkage strain at different ages and various factors under 60% RH.

Figure 7 depicts the relationship between the average drying shrinkage strain at different ages and various factors under 60% RH. Based on the average value K_avg_, it was observed that similar trends about the drying shrinkage strain at different ages were presented, which was consistent with those observed at 30% RH, while there was still a difference in the trend in the W/B ratio, presenting the minimum shrinkage strain at 0.38.

Based on the range analysis in Table 9 and Figure 7, the key factors were determined to be the FA/GGBS ratio and alkali content. As for the EGC specimens under 60% RH, according to the minimum shrinkage strain, the optimal FA/GGBS ratio, alkali content, W/B ratio, and fiber volume fraction were determined to be 0/100, 4%, 0.38, and 1.5%, respectively. This corresponded to the A_1_B_1_C_4_D_2_ group.

(3) 100% relative humidity

**Figure 8:**
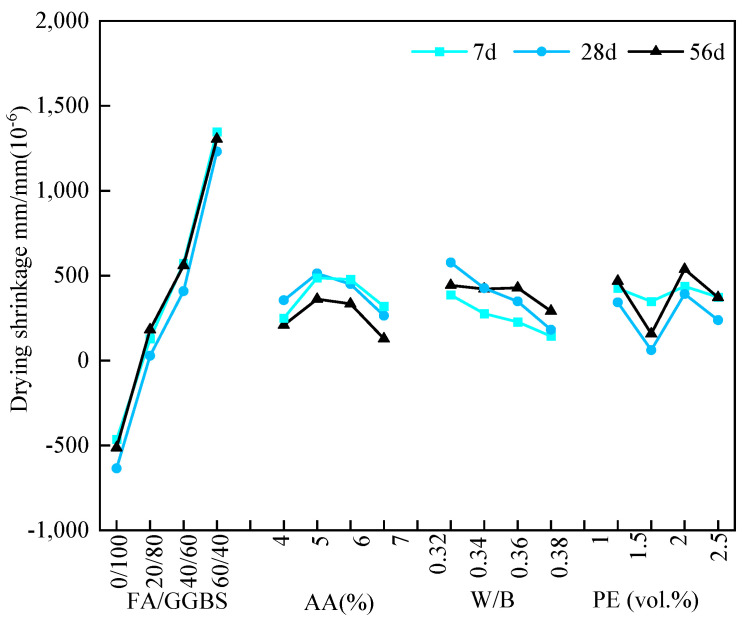
Relationship between the average shrinkage strain at different ages and various factors under 100% RH.

The results of the range analysis on the shrinkage strain of EGC specimens under 100% RH are presented in Table 10. According to the magnitude of R values, the key factor influencing the shrinkage strain of EGC specimens at 7 d, 28 d, and 56 d was the FA/GGBS ratio. And the other three factors had less influence. Figure 8 depicts the relationship between the average shrinkage strain at different ages and various factors under 100% RH. Based on the average value K_avg_, it was observed that the shrinkage strain at different ages increased with a higher FA/GGBS ratio, while an opposite phenomenon was observed on the K_avg_ value of the W/B ratio. Additionally, for an FA/GGBS ratio of 0/100, expansive behavior occurred in the EGC specimens. In a word, lower shrinkage strain was observed compared to those of the EGC specimens under 30% and 60% RH.

Based on the range analysis in Table 10 and Figure 8, the key factor was determined to be the FA/GGBS ratio. As for the EGC specimens under 100% RH, according to the minimum shrinkage strain, the optimal FA/GGBS ratio, alkali content, W/B ratio, and fiber volume fraction were determined to be 20/80, 7%, 0.38, and 1.5%, respectively. This corresponded to the A_2_B_4_C_4_D_2_ group.

### 3.3. Effect of Ambient Curing Humidity on Shrinkage Strain

**Figure 9:**
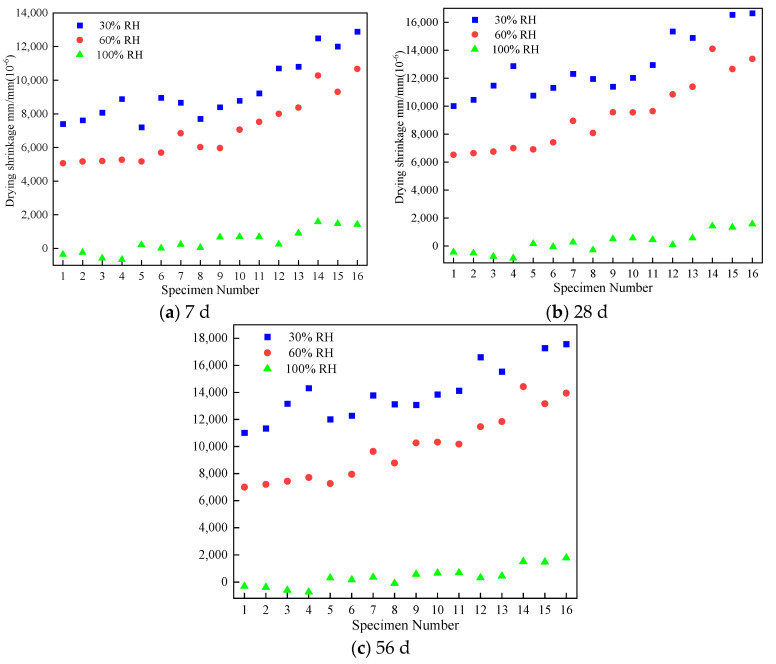
Shrinkage strain of each group at different ages under various ambient curing humidity values.

Figure 9 shows the shrinkage strain of each group at different ages under various ambient curing humidity values. It was clearly found that the shrinkage strain increased with the decrease in ambient curing humidity at all curing ages, presenting the highest shrinkage value under 30% RH and the lowest shrinkage value under 100% RH. Additionally, some data were below zero when EGC specimens were under 100% RH, indicating that expansive behavior occurred. To some extent, increasing ambient curing humidity was an effective method to reduce the shrinkage and shrinkage cracking. This phenomenon was mainly due to the high internal relative humidity, resulting in the reduction in capillary pore negative pressure [38,39].

Combined with the shrinkage data in Table 5, Table 6 and Table 7, the shrinkage strain reduction percentages of EGC specimens at 7 d, 28 d, and 56 d are shown in Table 11, Table 12, and Table 13, respectively. From Table 11, compared to the shrinkage strain under 30% RH at 7 d, the shrinkage strain values under 60% RH and 100% RH were reduced by 18.4%~40.7% and 88.9%~107.5%, respectively. From Table 12 and Table 13, for EGC specimens under 100% RH, the reduction percentage of shrinkage strain was close to that of specimens under 30% RH. And this reduction percentage was higher at 28 d and 56 d, presenting the highest values of about 45.1% and 46.1%, respectively.

### 3.4. Relationship between Shrinkage Strain and Curing Age

**Figure 10:**
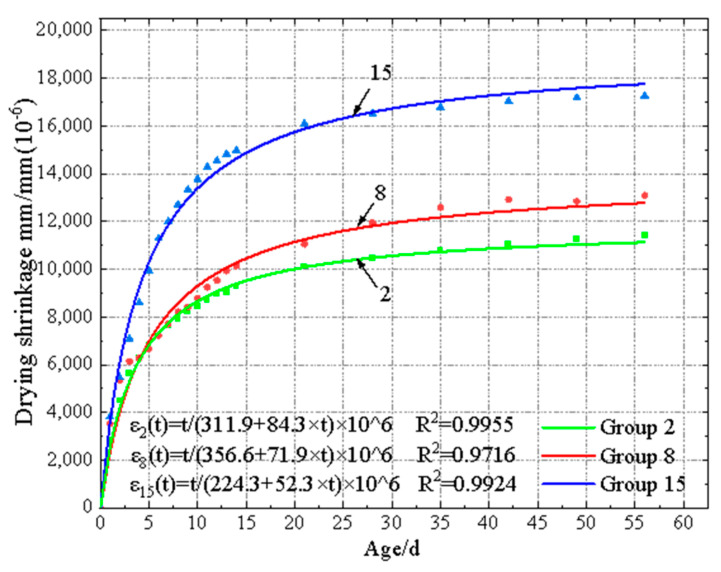
Shrinkage strain–curing age fitting curves of EGC specimens under 30% RH.

**Figure 11:**
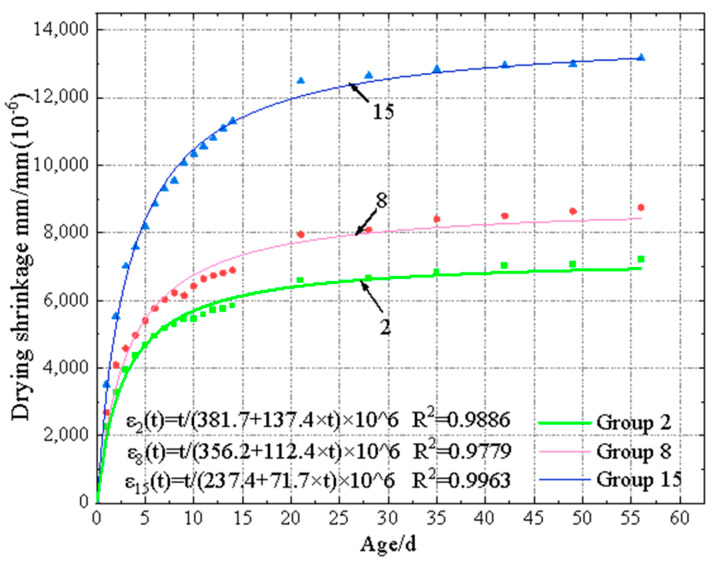
Shrinkage strain–curing age fitting curves of EGC specimens under 60% RH.

Shrinkage strain–curing age fitting curves of EGC specimens under 30% RH and 60% RH are shown in Figure 10 and Figure 11, respectively. Regression analysis was conducted based on the measured data, and a mathematical model was fitted to predict the shrinkage strain. According to the concrete drying shrinkage prediction model summarized in prior research [40], it was found that the proposed hyperbolic function could effectively fit the relationship between shrinkage strain and curing age, with the fitting equation shown in Equation (3).

(3) $$\varepsilon \left(t\right)=\frac{t\times 10^{6}}{a+b\times t}$$

where *ε*(*t*) represents the dry shrinkage strain of EGC at time t, and a and b are parameters related to the binder components. The parameters for each group’s fitting curve equation are listed in Table 14 and Table 15. From the data in these tables, it can be observed that under 30% RH, the fitting degree was from 0.9642 to 0.9987, while it was from 0.9492 to 0.9966 under 60% RH. It should be noted that there was no clear relationship between shrinkage strain and curing age, due to the appearance of expansive and shrinkage behavior.

## 4. Conclusions

This paper primarily investigated the drying shrinkage strain of EGC specimens under three different ambient curing relative humidity values. Sixteen specimens were prepared and tested through orthogonal experimental design and orthogonal experimental analysis methods. Four variables, namely, the FA/GGBS ratio, alkali content, W/B ratio, and fiber volume content, were analyzed. The variation law, key influencing factors, and the optimal mix design of EGC drying shrinkage strain under 30%, 60%, and 100% RH were obtained. And fitting equations between the shrinkage strain and curing age were established. The main conclusions could be summarized as follows:

(1) The drying shrinkage strain was decreased with the increase in ambient relative humidity. For specimens under 30% and 60% RH, the shrinkage strain developed rapidly at 7 d, with more than 55% of 56 d shrinkage strain.
(2) According to the range analysis, for specimens under 30% and 60% RH, the key factors affecting the drying shrinkage strain of EGC specimens under 30% and 60% RH were the FA/GGBS ratio and the alkali content. The optimal mix ratio was A_1_B_1_C_x_D_2_, with the FA/GGBS ratio, alkali content, and fiber volume fraction being 0/100, 4%, and 1.5%, respectively.
(3) For specimens under 30% and 60% RH, the decrease in slag content and increase in alkali content both resulted in greater drying shrinkage. The addition of fibers effectively reduced the shrinkage strain, while a minor impact on shrinkage was observed for the W/B ratio.
(4) Compared with the shrinkage strain under 30% RH, the reduction percentages of shrinkage strain under 60% and 100%RH were up to 46.1% and 107.5%, respectively.
(5) A relationship between shrinkage strain and curing age under 30% and 60% RH was established with a fitting degree from 0.9492 to 0.9987. No clear relationship was observed under 100% RH due to the appearance of expansive and shrinkage behavior.

This study points out that the increase in ambient relative humidity can significantly reduce the contraction strain of EGCs. A high humidity environment should be maintained during its early curing phase to ensure the good application of EGCs in engineering. In addition, the effect of ambient relative humidity on the shrinkage mechanism of EGCs should be further studied in future work.

## Figures and Tables

**Table 1:** Chemical composition of FA and GGBS.

Composition	SiO_2_	Al_2_O_3_	Fe_2_O_3_	CaO	MgO	Na_2_O
FA (%)	53.97	31.15	4.16	4.01	1.01	0.89
GGBS (%)	32.15	17.00	0.4	35.34	9.95	0.59

**Table 2:** Technical specifications of anhydrous sodium metasilicate.

Na_2_O Content	SiO_2_ Content	Particle Size	Density	Insoluble in Water
50.63%	46.27%	99.49 mesh	1.23 g/cm^3^	0.007%

**Table 3:** Technical specifications of PE fibers.

Diameter (μm)	Density (g/cm^3^)	Length (mm)	Tensile Strength (MPa)	Elastic Modulus (GPa)	Break Elongation (%)
19–43	0.98	12	3000	110	2–3

**Table 4:** Orthogonal table for EGC experiments.

Number		FA/GGBS Ratio	Alkali Content (%)	W/B Ratio	Fiber Content (vol.%)	Compressive Strength (MPa)
1	$A_{1}B_{1}C_{1}D_{1}$	0/100	4%	0.32	1.0%	78.6 ± 2.3
2	$A_{1}B_{2}C_{2}D_{2}$	0/100	5%	0.34	1.5%	77.5 ± 2.5
3	$A_{1}B_{3}C_{3}D_{3}$	0/100	6%	0.36	2.0%	75.0 ± 3.1
4	$A_{1}B_{4}C_{4}D_{4}$	0/100	7%	0.38	2.5%	75.7 ± 2.7
5	$A_{2}B_{1}C_{2}D_{3}$	20/80	4%	0.34	2.0%	67.3 ± 4.5
6	$A_{2}B_{2}C_{1}D_{4}$	20/80	5%	0.32	2.5%	82.3 ± 3.2
7	$A_{2}B_{3}C_{4}D_{1}$	20/80	6%	0.38	1.0%	71.8 ± 2.2
8	$A_{2}B_{4}C_{3}D_{2}$	20/80	7%	0.36	1.5%	68.7 ± 2.4
9	$A_{3}B_{1}C_{3}D_{4}$	40/60	4%	0.36	2.5%	53.5 ± 2.5
10	$A_{3}B_{2}C_{4}D_{3}$	40/60	5%	0.38	2.0%	59.8 ± 3.8
11	$A_{3}B_{3}C_{1}D_{2}$	40/60	6%	0.32	1.5%	66.4 ± 1.8
12	$A_{3}B_{4}C_{2}D_{1}$	40/60	7%	0.34	1.0%	81.7 ± 4.1
13	$A_{4}B_{1}C_{4}D_{2}$	60/40	4%	0.38	1.5%	42.8 ± 2.1
14	$A_{4}B_{2}C_{3}D_{1}$	60/40	5%	0.36	1.0%	62.8 ± 3.2
15	$A_{4}B_{3}C_{2}D_{4}$	60/40	6%	0.34	2.5%	60.0 ± 2.7
16	$A_{4}B_{4}C_{1}D_{3}$	60/40	7%	0.32	2.0%	65.3 ± 2.3

**Table 5:** Dry shrinkage strain of EGC specimens at 7 d, 28 d, and 56 d under 30% RH.

Specimen Number		Drying Shrinkage Strain (10^−6^)		
		7 d	28 d	56 d
1	A_1_B_1_C_1_D_1_	7398	10,021	11,017
2	A_1_B_2_C_2_D_2_	7620	10,462	11,342
3	A_1_B_3_C_3_D_3_	8076	11,476	13,167
4	A_1_B_4_C_4_D_4_	8886	12,876	14,320
5	A_2_B_1_C_2_D_3_	7200	10,768	12,007
6	A_2_B_2_C_1_D_4_	8951	11,319	12,281
7	A_2_B_3_C_4_D_1_	8663	12,324	13,784
8	A_2_B_4_C_3_D_2_	7705	11,946	13,131
9	A_3_B_1_C_3_D_4_	8395	11,397	13,076
10	A_3_B_2_C_4_D_3_	8778	12,035	13,856
11	A_3_B_3_C_1_D_2_	9224	12,954	14,128
12	A_3_B_4_C_2_D_1_	10,707	15,349	16,595
13	A_4_B_1_C_4_D_2_	10,802	14,882	15,539
14	A_4_B_2_C_3_D_1_	12,492	18,600	19,472
15	A_4_B_3_C_2_D_4_	12,002	16,536	17,266
16	A_4_B_4_C_1_D_3_	12,889	16,656	17,563

**Table 6:** Dry shrinkage strain of EGC specimens at 7 d, 28 d, and 56 d under 60% RH.

Specimen Number		Drying Shrinkage Strain (10^−6^)		
		7 d	28 d	56 d
1	A_1_B_1_C_1_D_1_	5067	6529	6998
2	A_1_B_2_C_2_D_2_	5170	6646	7202
3	A_1_B_3_C_3_D_3_	5200	6756	7435
4	A_1_B_4_C_4_D_4_	5272	7008	7716
5	A_2_B_1_C_2_D_3_	5172	6916	7263
6	A_2_B_2_C_1_D_4_	5692	7416	7948
7	A_2_B_3_C_4_D_1_	6851	8956	9639
8	A_2_B_4_C_3_D_2_	6022	8088	8785
9	A_3_B_1_C_3_D_4_	5969	9569	10,268
10	A_3_B_2_C_4_D_3_	7063	9560	10,327
11	A_3_B_3_C_1_D_2_	7524	9651	10,180
12	A_3_B_4_C_2_D_1_	8005	10,851	11,465
13	A_4_B_1_C_4_D_2_	8376	11,392	11,848
14	A_4_B_2_C_3_D_1_	10,276	14,103	14,419
15	A_4_B_3_C_2_D_4_	9307	12,659	13,164
16	A_4_B_4_C_1_D_3_	10,677	13,384	13,944

**Table 7:** Dry shrinkage strain of EGC specimens at 7 d, 28 d, and 56 d under 100% RH.

Specimen Number		Drying Shrinkage Strain (10^−6^)		
		7 d	28 d	56 d
1	A_1_B_1_C_1_D_1_	−361	−432	−319
2	A_1_B_2_C_2_D_2_	−245	−503	−396
3	A_1_B_3_C_3_D_3_	−588	−748	−607
4	A_1_B_4_C_4_D_4_	−664	−860	−735
5	A_2_B_1_C_2_D_3_	213	169	304
6	A_2_B_2_C_1_D_4_	16	−51	163
7	A_2_B_3_C_4_D_1_	230	280	356
8	A_2_B_4_C_3_D_2_	47	−288	−95
9	A_3_B_1_C_3_D_4_	667	516	576
10	A_3_B_2_C_4_D_3_	695	572	663
11	A_3_B_3_C_1_D_2_	684	456	679
12	A_3_B_4_C_2_D_1_	245	89	317
13	A_4_B_1_C_4_D_2_	902	578	439
14	A_4_B_2_C_3_D_1_	1585	1429	1516
15	A_4_B_3_C_2_D_4_	1472	1347	1480
16	A_4_B_4_C_1_D_3_	1429	1570	1786

**Table 8:** Results of the range analysis on the drying shrinkage strain of EGC specimens under 30% RH.

Age	Indicator	Level	Factors			
			FA/GGBS Ratio	Alkali Content (%)	W/B Ratio	Fiber Content (vol.%)
7 d	K_avg_ (10^−6^)	1	7995	8449	9616	9815
		2	8130	9460	9382	8838
		3	9276	9491	9167	9236
		4	12,046	10,047	9282	9559
	R (10^−6^)		4051	1598	449	977
	Optimal Level		1	1	3	2
28 d	K_avg_ (10^−6^)	1	11,209	11,767	12,738	14,074
		2	11,589	13,104	13,279	12,561
		3	12,934	13,323	13,355	12,734
		4	16,669	14,207	13,029	13,032
	R (10^−6^)		5460	2440	617	1513
	Optimal Level		1	1	1	2
56	K_avg_ (10^−6^)	1	12,462	12,910	13,747	15,217
		2	12,801	14,238	14,303	13,535
		3	14,414	14,586	14,712	14,148
		4	17,460	15,402	14,375	14,236
	R (10^−6^)		4999	2493	964	1682
	Optimal Level		1	1	1	2

Note: K_avg_ refers to the average drying shrinkage strain of four test specimens under different levels of the corresponding factors, representing the average effect value of the factor and its level. R is the range of K_avg_ values across the four levels of the corresponding factor, indicating the extent of the factor’s impact on shrinkage strain.

**Table 9:** Results of the range analysis on the drying shrinkage strain of EGC specimens under 60% RH.

Age	Indicator	Level	Factors			
			FA/GGBS Ratio	Alkali Content (%)	W/B Ratio	Fiber Content (vol.%)
7 d	K_avg_ (10^−6^)	1	5177	6146	7240	7550
		2	5934	7050	6914	6773
		3	7140	7220	6867	7028
		4	9659.0	7494	6890	6560
	R (10^−6^)		4482	1348	373	990
	Optimal Level		1	1	4	4
28 d	K_avg_ (10^−6^)	1	6735	8602	9245	10,110
		2	7844	9431	9268	8944
		3	9908	9505	9629	9154
		4	12,884	9833	9229	9163
	R (10^−6^)		6150	1231	400	1165
	Optimal Level		1	1	4	2
56	K_avg_ (10^−6^)	1	7338	9094	9768	10,630
		2	8409	9974	9774	9504
		3	10,560	10,105	10227	9742
		4	13,344	10,478	9883	9774
	R (10^−6^)		6006	1383	459	1127
	Optimal Level		1	1	1	2

**Table 10:** Results of the range analysis on the shrinkage strain of EGC specimens under 100% RH.

Age	Indicator	Level	Factors			
			FA/GGBS Ratio	Alkali Content (%)	W/B Ratio	Fiber Content (vol.%)
7 d	K_avg_ (10^−6^)	1	−465	355	442	425
		2	127	513	421	347
		3	573	450	428	437
		4	1347	264	291	373
	R (10^−6^)		1812	248	151	91
	Optimal Level		2	4	4	2
28 d	K_avg_ (10^−6^)	1	−636	208	386	342
		2	28	362	276	61
		3	408	334	227	391
		4	1231	128	143	238
	R (10^−6^)		1867	234	243	330
	Optimal Level		2	4	4	2
56	K_avg_ (10^−6^)	1	−514	250	577	468
		2	182	486	426	157
		3	559	477	348	537
		4	1305	318	181	371
	R (10^−6^)		1819	236	396	380
	Optimal Level		2	1	4	2

**Table 11:** Shrinkage strain reduction percentage of EGC specimens at 7 d.

Specimen Number		Shrinkage Strain Reduction Percentage (%)	
		${(\varepsilon }_{30\%RH}-\varepsilon_{60\%RH})\times 100\%/\varepsilon_{30\%RH}$	${(\varepsilon }_{30\%RH}-\varepsilon_{100\%RH})\times 100\%/\varepsilon_{30\%RH}$
1	A_1_B_1_C_1_D_1_	31.5	104.9
2	A_1_B_2_C_2_D_2_	32.1	103.2
3	A_1_B_3_C_3_D_3_	35.6	107.3
4	A_1_B_4_C_4_D_4_	40.7	107.5
5	A_2_B_1_C_2_D_3_	28.2	97.0
6	A_2_B_2_C_1_D_4_	36.4	99.8
7	A_2_B_3_C_4_D_1_	20.9	97.4
8	A_2_B_4_C_3_D_2_	21.8	99.4
9	A_3_B_1_C_3_D_4_	28.9	92.1
10	A_3_B_2_C_4_D_3_	19.5	92.1
11	A_3_B_3_C_1_D_2_	18.4	92.6
12	A_3_B_4_C_2_D_1_	25.2	97.7
13	A_4_B_1_C_4_D_2_	22.5	91.7
14	A_4_B_2_C_3_D_1_	17.7	87.3
15	A_4_B_3_C_2_D_4_	22.5	87.7
16	A_4_B_4_C_1_D_3_	17.2	88.9

**Table 12:** Shrinkage strain reduction percentage of EGC specimens at 28 d.

Specimen Number		Shrinkage Strain Reduction Percentage (%)	
		${(\varepsilon }_{30\%RH}-\varepsilon_{60\%RH})\times 100\%/\varepsilon_{30\%RH}$	${(\varepsilon }_{30\%RH}-\varepsilon_{100\%RH})\times 100\%/\varepsilon_{30\%RH}$
1	A_1_B_1_C_1_D_1_	34.9	104.3
2	A_1_B_2_C_2_D_2_	36.5	104.8
3	A_1_B_3_C_3_D_3_	41.1	106.5
4	A_1_B_4_C_4_D_4_	45.6	106.7
5	A_2_B_1_C_2_D_3_	35.8	98.4
6	A_2_B_2_C_1_D_4_	34.5	100.5
7	A_2_B_3_C_4_D_1_	27.3	97.7
8	A_2_B_4_C_3_D_2_	32.3	102.4
9	A_3_B_1_C_3_D_4_	16.0	95.5
10	A_3_B_2_C_4_D_3_	20.6	95.3
11	A_3_B_3_C_1_D_2_	25.5	96.5
12	A_3_B_4_C_2_D_1_	29.3	99.4
13	A_4_B_1_C_4_D_2_	23.5	96.1
14	A_4_B_2_C_3_D_1_	24.2	92.3
15	A_4_B_3_C_2_D_4_	23.5	91.9
16	A_4_B_4_C_1_D_3_	19.6	90.6

**Table 13:** Shrinkage strain reduction percentage of EGC specimens at 56 d.

Specimen Number		Shrinkage Strain Reduction Percentage (%)	
		${(\varepsilon }_{30\%RH}-\varepsilon_{60\%RH})\times 100\%/\varepsilon_{30\%RH}$	${(\varepsilon }_{30\%RH}-\varepsilon_{100\%RH})\times 100\%/\varepsilon_{30\%RH}$
1	A_1_B_1_C_1_D_1_	36.5	102.9
2	A_1_B_2_C_2_D_2_	36.5	103.5
3	A_1_B_3_C_3_D_3_	43.5	104.6
4	A_1_B_4_C_4_D_4_	46.1	105.1
5	A_2_B_1_C_2_D_3_	39.5	97.5
6	A_2_B_2_C_1_D_4_	35.3	98.7
7	A_2_B_3_C_4_D_1_	30.1	97.4
8	A_2_B_4_C_3_D_2_	33.1	100.7
9	A_3_B_1_C_3_D_4_	21.5	95.6
10	A_3_B_2_C_4_D_3_	25.5	95.2
11	A_3_B_3_C_1_D_2_	27.9	95.2
12	A_3_B_4_C_2_D_1_	30.9	98.1
13	A_4_B_1_C_4_D_2_	23.8	97.2
14	A_4_B_2_C_3_D_1_	26.0	92.2
15	A_4_B_3_C_2_D_4_	23.8	91.4
16	A_4_B_4_C_1_D_3_	20.6	89.8

**Table 14:** Regression equation parameters for the shrinkage strain–curing age fitting curves under 30% RH.

RH	Number	Regression Equation	a	b	R^2^
30%	1	$\varepsilon \left(t\right)=\frac{t\times 10^{6}}{a+b\times t}$	185.6	73.9	0.9943
	2		311.9	84.3	0.9955
	3		387.1	86.3	0.9961
	4		312.1	66.1	0.9962
	5		165.5	71.0	0.9857
	6		227.2	78.4	0.9987
	7		278.6	70.8	0.9762
	8		356.6	71.9	0.9716
	9		258.7	45.0	0.9703
	10		92.0	47.0	0.9642
	11		270.1	68.1	0.9862
	12		249.7	56.7	0.9914
	13		249.2	58.1	0.9955
	14		207.7	46.9	0.9939
	15		224.3	52.3	0.9924
	16		185.8	53.2	0.9980

**Table 15:** Regression equation parameters for the shrinkage strain–curing age fitting curves under 60% RH.

RH	Number	Regression Equation	a	b	R^2^
60%	1	$\varepsilon \left(t\right)=\frac{t\times 10^{6}}{a+b\times t}$	202.7	106.7	0.9966
	2		381.7	137.4	0.9886
	3		382.0	134.9	0.9852
	4		398.6	129.5	0.9804
	5		86.9	75.5	0.9930
	6		292.8	126.9	0.9706
	7		251.5	104.5	0.9719
	8		356.2	112.4	0.9779
	9		669.5	81.9	0.9492
	10		82.3	59.9	0.9867
	11		232.3	97.5	0.9830
	12		242.2	86.2	0.9702
	13		263.7	79.7	0.9952
	14		212.7	63.4	0.9900
	15		237.4	71.7	0.9963
	16		190.4	67.1	0.9947

## Data Availability

The raw data supporting the conclusions of this article will be made available by the authors on request.
